# Characterization and Hydrolysis Studies of a Prodrug Obtained as Ester Conjugate of Geraniol and Ferulic Acid by Enzymatic Way

**DOI:** 10.3390/ijms25116263

**Published:** 2024-06-06

**Authors:** Lindomar Alberto Lerin, Giada Botti, Alessandro Dalpiaz, Anna Bianchi, Luca Ferraro, Chaimae Chaibi, Federico Zappaterra, Domenico Meola, Pier Paolo Giovannini, Barbara Pavan

**Affiliations:** 1 Department of Chemical, Pharmaceutical and Agricultural Sciences, University of Ferrara, Via Luigi Borsari, 46, I-44121 Ferrara, Italy; lrnldm@unife.it (L.A.L.); bttgdi@unife.it (G.B.); bna@unife.it (A.B.); chaimae.chaibi@edu.unife.it (C.C.); zppfrc@unife.it (F.Z.); domenico.meola@unife.it (D.M.); gvnppl@unife.it (P.P.G.); 2 Center for Translational Neurophysiology of Speech and Communication (CTNSC@UniFe), Italian Institute of Technology (IIT), Via Fossato di Mortara 19, I-44121 Ferrara, Italy; pvnbbr@unife.it; 3 Department of Life Sciences and Biotechnology, University of Ferrara and LTTA Center, Via Fossato di Mortara 19, I-44121 Ferrara, Italy; frl@unife.it; 4 Department of Neuroscience and Rehabilitation—Section of Physiology, University of Ferrara, Via L. Borsari 46, I-44121 Ferrara, Italy

**Keywords:** geraniol, ferulic acid, bio-catalyzed, prodrug, solid lipid microparticles, N2a cells, oxidative injury

## Abstract

Ferulic acid (Fer) and geraniol (Ger) are natural compounds whose antioxidant and anti-inflammatory activity confer beneficial properties, such as antibacterial, anticancer, and neuroprotective effects. However, the short half-lives of these compounds impair their therapeutic activities after conventional administration. We propose, therefore, a new prodrug (Fer-Ger) obtained by a bio-catalyzed ester conjugation of Fer and Ger to enhance the loading of solid lipid microparticles (SLMs) designed as Fer-Ger delivery and targeting systems. SLMs were obtained by hot emulsion techniques without organic solvents. HPLC-UV analysis evidenced that Fer-Ger is hydrolyzed in human or rat whole blood and rat liver homogenates, with half-lives of 193.64 ± 20.93, 20.15 ± 0.75, and 3.94 ± 0.33 min, respectively, but not in rat brain homogenates. Studies on neuronal-differentiated mouse neuroblastoma N2a cells incubated with the reactive oxygen species (ROS) inductor H_2_O_2_ evidenced the Fer-Ger ability to prevent oxidative injury, despite the fact that it appears ROS-promoting. The amounts of Fer-Ger encapsulated in tristearin SLMs, obtained in the absence or presence of glucose, were 1.5 ± 0.1%, allowing the control of the prodrug release (glucose absence) or to sensibly enhance its water dissolution rate (glucose presence). These new “green” carriers can potentially prolong the beneficial effects of Fer and Ger or induce neuroprotection as nasal formulations.

## 1. Introduction

Natural compounds are currently considered a potential source for novel therapeutic agents. For example, efficacious antibacterial, anticancer, or neuroprotective activities were evidenced for some polyphenols [1,2,3] and main components of essential oils [4,5,6]. Among the polyphenols, ferulic acid (Fer) was proposed for the management of diabetes and its complications [7] or to control cancer progression [2]. Moreover, the usefulness of Fer against neurodegenerative diseases has been recognized [8]. The peripheral and central effects of this polyphenol are mainly attributed to its radical scavenging and antioxidant properties, along with its anti-inflammatory activity [8,9]. Similar features were also attributed to geraniol (Ger), which, among the main components of essential oils, appears to be able to induce anticancer effects [10] and neuroprotection [11], in particular as far as Parkinson’s disease is concerned [12]. The combined administration of Fer and Ger may therefore be promising for the prevention and therapy of diabetes, cancer, and neurodegenerative diseases. High oral bioavailability and the ability to permeate the central nervous system (CNS) characterize these compounds [13,14,15,16], suggesting that their oral administration may allow to obtain their beneficial effects at both peripheral and central levels. On the other hand, both Ger and Fer are characterized by very short in vivo half-lives, ranging from a few minutes to 30 min in rodents and humans [14,17,18,19]. Therefore, after administration as conventional formulations, the permanence of these compounds in the bloodstream and at the central level appears poor for therapeutic purposes [18,19,20]. New formulations have been recently proposed to overcome this issue, with the general aim of maximizing Fer and/or Ger targeting at their therapeutic sites. In particular, micro-emulsified systems, or micro- and nano-particles based on polymers or lipids, were formulated to increase the Fer central effects [18,21,22,23] and its anticancer activity [24]. Alternatively, the prodrug strategy has been demonstrated to be promising in enhancing the Fer ability to cross the blood–brain barrier or in promoting its antitumor activity at the intestinal level [24]. Concerning the essential oils, polymer-based hydrogels were proposed as carriers for the treatment of infected wounds [4], whereas an emulsified formulation of Ger obtained by using the amphiphilic polymer chitosan-oleate increased its ability to reach the CNS [16].

**Figure 1:**
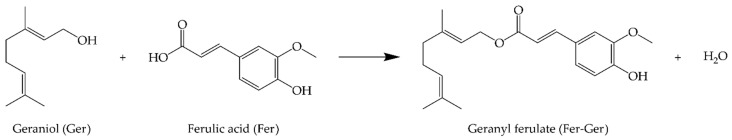
Geranyl ferulate (Fer-Ger) obtained by the ester conjugation of ferulic acid (Fer) and geraniol (Ger).

We have recently proposed the prodrug strategy for both Ger and Fer to increase their encapsulation efficiency in polymeric or lipid particulate systems, promoting their controlled release [25] or brain targeting [26,27]. In this regard, the prodrug synthesis appeared particularly useful to prevent the high Ger volatility that normally hampers its encapsulation [27]. Considering these aspects, in the present work we propose the synthesis and characterization of a new prodrug (Fer-Ger, Figure 1) obtained by the bio-catalyzed esterification of the natural compounds Ger and Fer. The enzymatic reactions have been performed either under solvent-free conditions or with the aid of an organic solvent. The Fer-Ger obtained has been purified and characterized, as summarized below.

The behavior of this conjugate as a prodrug was characterized by analyzing its aptitude to be hydrolyzed in physiological environments, such as human and rat whole blood, or rat liver and brain homogenates. Moreover, the potential retention of the antioxidant properties of Ger and Fer upon conjugation was investigated by analyzing the potential protective effects of Fer-Ger against oxidative stress on neuronal cells. Taking into account that the efficacy or toxicity of the drugs can be related to their intracellular concentrations [28], Fer-Ger uptake studies in neuronal cells were also performed. Finally, in order to obtain carriers potentially able to induce a prolonged release of Fer-Ger or its targeting in the CNS, solid lipid microparticles (SLMs) were formulated and characterized as far as conjugate loading and release are concerned. SLMs were selected as Fer-Ger carriers because they can be obtained in the absence of organic solvent by using biocompatible and biodegradable excipients, or, in other words, with procedures coherent with the low environmental impact related to the synthesis of the conjugate.

## 2. Results and Discussion

### 2.1. Fer-Ger Enzymatic Synthesis

Despite the apparent simplicity of the Ger and Fer condensation, to the best of our knowledge, the synthesis of Fer-Ger has been reported in only two articles. Searching from a selective method for the production of a Ger esters library, García Santos et al. [29] obtained the target compound in 10% isolated yield through the condensation of Ger and Fer catalyzed by Gd(TfO)_3_. In addition to the low yield, this method also suffers from a low atom economy because of the super-stoichiometric amounts of I_2_, PPh_3_, and imidazole (or *N*-methylimidazole) necessary for the acid’s activation. Furthermore, the use of a chlorinated solvent (CH_2_Cl_2_) is also needed. A more sustainable synthetic strategy has been reported by Katsoura et al. [30], who obtained Fer-Ger in 36% yield through the enzymatic esterification of Fer with Ger performed in the ionic liquid [bmim]PF_6_ (1-butyl-3-methylimidazolium hexafluorophosphate).

To develop a sustainable synthetic strategy that allows the preparation of Fer-Ger with higher yields, we moved to investigate the reaction conditions for the enzyme-catalyzed condensation of Fer and Ger. The biocatalysts Lipozyme^®^ 435 (following indicated as Lipo-435) and the lipase from *Rhizmucor miehei* Lipozyme^®^ RM IM (following indicated as Lipo-RM) have been chosen based on what was previously reported by Katsoura et al. [30], while an accurate study has been dedicated to identifying the best reaction solvent and, as an alternative, the feasibility of the reaction under solvent-free conditions. Organic solvents play a crucial role in esterification reactions catalyzed by lipases in non-aqueous media. These solvents can influence mass transfer in the reaction system by modifying the solubility of the substrates. Additionally, they significantly influence the lipase’s structure, affecting the activity and stability of the enzyme. The partition coefficient (Log *P*) is the ratio between the concentrations of a compound in a mixture of two immiscible phases, consisting of 1-octanol and water, at equilibrium. It is primarily used to describe the solvent’s hydrophobicity. Based on Log *P*, organic solvents can be classified as hydrophilic—Log *P* < 1.4; medium hydrophilic—Log *P* > 1.4 and < 3.5; and hydrophobic—Log *P* > 3.5. Therefore, Log *P* is a key factor influencing substrates’ relative solubility, activity, and specificity in an enzymatic reaction [31,32].

**Figure 2:**
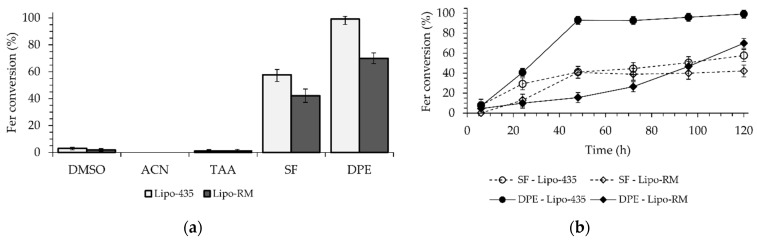
Enzymatic esterification of Fer with Ger: (**a**) effect of the presence and kind of reaction solvent and (**b**) time-course of the reaction catalyzed by Lipo-435 or Lipo-RM in a solvent-free system or DPE as the reaction solvent. The reaction conditions are reported in Section 3.3.

This study utilized five organic solvents, including Ger, which served as the substrate and the solvent (Figure 2a). The candidate solvents had Log *P* values ranging from −1.30 to 4.21. Dimethylsulfoxide (DMSO) with a Log *P* of −1.30, CH_3_CN with a Log *P* of −0.33, and *t*-amyl alcohol (TAA) with a Log *P* of 0.89 [33] exhibited the highest solubilization of Fer and the lowest hydrophobicity among all the solvents used. The enzymatic reactions performed in DMSO and TAA demonstrated less than 2% conversions, whereas no conversion of Fer to Fer-Ger was observed in CH_3_CN after 120 h for both enzymes. On the other hand, solvents with greater hydrophobicity and lower solubilization of Fer, such as Ger with a Log *P* of 3.56 [34] and diphenyl ether (DPE) with a Log *P* of 4.21 [35], allowed conversions of 58% and 99% for the reaction catalyzed by the Lipo-435 and of 43% and 70% for the ones performed with the Lipo-RM (Figure 2a).

The use of DPE resulted in higher conversions than the solvent-free system, with an increase of 41% and 15% observed for Lipo-435 and Lipo-RM, respectively. These results are worth noting if compared with those reported by Katsoura et al. [30], who used the same lipases and the ionic liquid [bmim]PF_6_ (Log *P* of −1.03) as the solvent and found that Lipo-RM had a higher conversion (36.1%) than Lipo-435 (only 6.7%) at a temperature of 60 °C over 72 h.

A time-course study was then performed to evaluate the kinetics of the reactions promoted by the two lipases either in a solvent-free system or in the presence of DPE as the solvent (Figure 2b). The reaction carried out with DPE and Lipo-435 showed a high conversion of 93% in just 48 h, reaching a maximum of 99% in 120 h. The reaction with Lipo-RM showed the slowest kinetic in the first 48 h, with a slight increase in the reaction rate in the subsequent period, reaching a maximum conversion of 70% after 120 h. In the solvent-free system, a similar kinetic behavior was observed for both lipases, with a higher reaction rate in the first 48 h, which allowed for a conversion rate of around 40%. However, after this initial period, there was an abrupt decrease in the reaction rate for both enzymes, which determined final conversions of 58% and 43% after 120 h for the reactions catalyzed by Lipo-435 and Lipo-RM, respectively (Figure 2b).

The results found in this study are in accordance with the literature, which reports that organic solvents with higher Log *P* (nonpolar) have a reduced ability to remove essential water from the enzyme structure. Therefore, the solvent interaction in the region that protects the catalytic site can increase catalytic activity since the lipase is maintained in a flexible, open, and active arrangement. Consequently, these characteristics make lipases more stable and active in high Log *P* solvents (Ger and DPE) [36,37]. The present study furnishes a straightforward methodology to synthesize Fer-Ger with a nearly quantitative yield by using the large-scale commercial Lipo-435 as the biocatalyst and DPE as the solvent. The same enzyme was found to be active under solvent-free conditions, allowing it to reach a maximum yield of 58%, which is higher than those reported in previous works [29,30].

### 2.2. Hydrolysis Studies of Fer-Ger

The first step of Fer-Ger characterization was the evaluation of its potential hydrolysis pattern in physiological environments in order to characterize its prodrug behavior. In particular, Fer-Ger was incubated in different media, such as Tris-HCl buffer (pH 7.4), human whole blood, rat whole blood, or rat brain and liver homogenates. Efficient extraction and analytical procedures were developed in order to quantify, at different time-points, Fer-Ger and its potential hydrolysis product Ger in all incubation media.

Fer-Ger was not degraded in Tris-HCl buffer (the medium used to obtain the homogenates) during its incubation at 37 °C for three hours, so any potential degradation observed for this compound in rat brain or liver homogenates cannot be attributed to the buffer solution.

**Figure 3:**
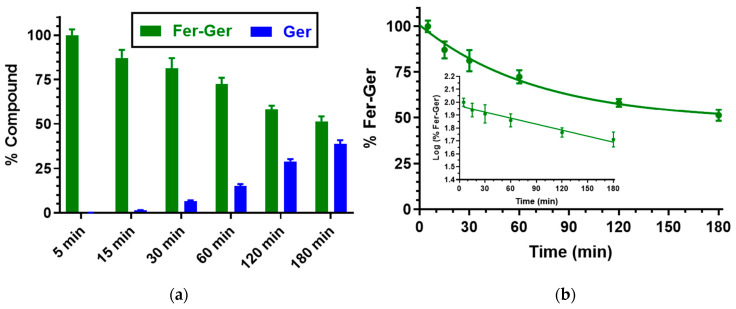
(**a**) Degradation profile of the prodrug Fer-Ger (green) and the corresponding appearance profile of its hydrolysis product Ger (blue) in human whole blood. All the values are reported as the percentage of the overall amount of incubated Fer-Ger. (**b**) Degradation profile of the prodrug Fer-Ger in human whole blood. The degradation of the prodrug follows pseudo-first-order kinetics, confirmed by the linearity of the semi-logarithmic plot reported in the inset (*n* = 6, *r* = 0.977, *p* < 0.001), whose half-life value is 193.64 ± 20.93 min. All data are reported as the mean ± S.E.M. of three independent experiments.

We have previously demonstrated that Ger is characterized by high stability in human and rat whole blood [19], or in rat liver and brain homogenates [27]. On the other hand, Fer-Ger was hydrolyzed in whole blood, evidencing significant rate differences between rat and human species. In particular, Figure 3 reports that in human whole blood Fer-Ger was degraded during time, allowing the appearance of its hydrolysis product Ger, whose amounts increased over time: after three hours of incubation in human whole blood, the remaining amount of Fer-Ger was about 50% of its overall incubated amount; its hydrolysis followed a pseudo-first order kinetic (half-life—t_1/2_ = 193.64 ± 20.93 min) confirmed by the linear pattern of the semi-logarithmic plot reported in the inset (*n* = 6, *r* = 0.977, *p* < 0.001). These data evidence that Fer-Ger can be considered a prodrug of Ger and Fer in human physiologic environments.

**Figure 4:**
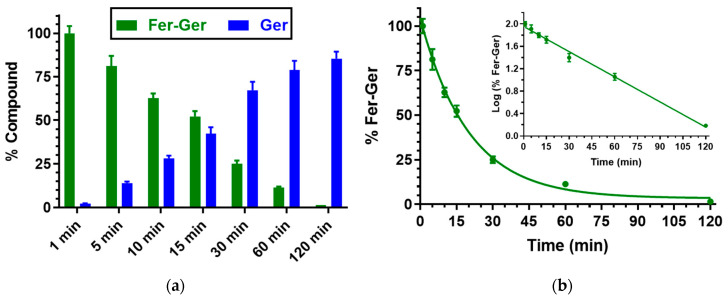
(**a**) Degradation profile of the prodrug Fer-Ger (green) and the corresponding appearance profile of Ger (blue) in rat whole blood. All the values are reported as the percentage of the overall amount of incubated Fer-Ger. (**b**) Degradation profile of the prodrug Fer-Ger in rat whole blood. The degradation of the prodrug follows a pseudo-first-order kinetic, confirmed by the linearity of the semi-logarithmic plot reported in the inset (*n* = 6, *r* = 0.997, *p* < 0.0001), whose half-life value is 20.15 ± 0.75 min. All data are reported as the mean ± S.E.M. of three independent experiments.

Besides human whole blood, even the potential hydrolysis of Fer-Ger in rat whole blood was investigated. In this case, as reported in Figure 4, Fer-Ger was almost completely degraded within 2 h, allowing the appearance of its hydrolysis product Ger with amounts increasing over time. The prodrug hydrolysis followed a pseudo-first-order kinetic (half-life—t_1/2_ = 20.15 ± 0.75 min) confirmed by the linear pattern of the semi-logarithmic plot reported in the inset (*n* = 6, *r* = 0.997, *p* < 0.0001). The hydrolysis rate appeared to be about 10 times faster than in human whole blood.

The higher hydrolysis rate of Fer-Ger detected in rat whole blood, in comparison to human blood, appears in good agreement with the results obtained by previous studies on the hydrolysis of a prodrug obtained by the ester conjugation of zidovudine (an antiviral agent) with ursodeoxycholic acid (UDCA-AZT) [38], evidencing that in comparison to humans, the blood of rodents has a stronger esterase activity, as previously described by other authors [39,40].

**Figure 5:**
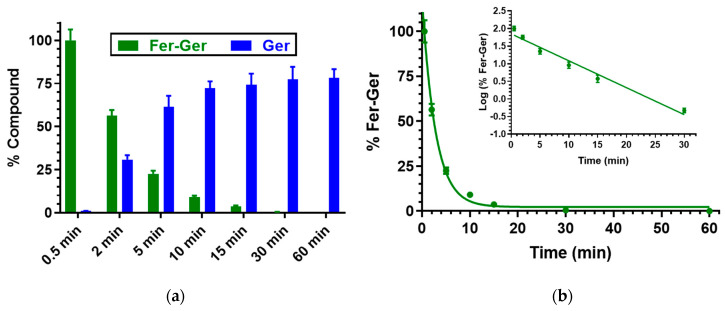
(**a**) Degradation profile of the prodrug Fer-Ger (green) and the corresponding appearance profile of Ger (blue) in rat liver homogenate. All the values are reported as the percentage of the overall amount of incubated Fer-Ger. (**b**) Degradation profile of the prodrug Fer-Ger in rat liver homogenate. The degradation of the prodrug a follows pseudo-first-order kinetic, confirmed by the linearity of the semi-logarithmic plot reported in the inset (*n* = 6, *r* = 0.986, *p* < 0.001), whose half-life value is 3.94 ± 0.33 min. All data are reported as the mean ± S.E.M. of three independent experiments.

Finally, the prodrug hydrolysis was confirmed also in rat liver homogenates, as reported in Figure 5, evidencing a pseudo-first-order kinetic, confirmed by the linearity of the semi-logarithmic plot reported in the inset (*n* = 6, *r* = 0.986, *p* < 0.001), with a half-life value of 3.94 ± 0.33 min.

In this case, about 30 min were enough to obtain the complete hydrolysis of the prodrug. A comparison with our previous results indicates that the hydrolysis rate of ester prodrugs in rat liver homogenates is dependent on the chemical species that have been conjugated. For example, the UDCA-AZT prodrug showed the same hydrolysis rate as Fer-Ger, whereas the half-life value of a prodrug obtained by the ester conjugation of Ger with ursodeoxycholic acid (GER-UDCA) was found to be higher than 30 min [27].

The in vitro hydrolysis experiments performed in whole blood and liver homogenates allowed us to characterize the prodrug behavior of Fer-Ger in peripheral compartments of a living organism, but not at the central level. In order to elucidate this aspect, Fer-Ger was also incubated in rat brain homogenates. In this case, the compound was characterized by high stability within 3 h, evidencing the total absence of hydrolysis processes. This behavior completely differs from those of other ester prodrugs that were hydrolyzed in rat brain homogenates, where, for example, UDCA-AZT evidenced a half-life value of about 7 min [38], whereas the hydrolysis of GER-UDCA was sensibly slower (about 30% hydrolyzed within 6 h) [27], similarly to a prodrug obtained by the ester dimerization of Fer (Fer-Fer, about 50% hydrolyzed within 6 h) [26]. Interestingly, the ester methylation of the prodrug Fer-Fer on the carboxylic moiety, which allowed to obtain a further Fer prodrug (Fer-Fer-Me), induced a strong increase in hydrolysis rate in rat brain homogenates (half-life value of about 14 min [26]). Surprisingly, a very simple prodrug of Fer, obtained by its esterification with methanol (Fer-Me), evidenced the same behavior of Fer-Ger in rat brain homogenates, i.e., the total absence of hydrolysis processes [25]. Overall, these results evidence that the hydrolysis rate of ester conjugates in physiologic environments is not dependent on their molecular size, but it is subject to sensibly changing based on the choice of molecules to conjugate.

Taking into account that Fer-Ger was not hydrolyzed in brain homogenates, its properties were further investigated, in particular by evaluating its potential toxicity or neuroprotective effects on neuronal cells.

### 2.3. Analysis of Fer-Ger Activity on Neuronal Differentiated Cells

Neuroblastoma N2a cells, i.e., cholinergic cells suitable to be differentiated into either cholinergic or dopaminergic brain cells depending on the culture conditions [41], have been used to evaluate the potential cytotoxicity of Fer-Ger and its feasible neuroprotective activity against H_2_O_2_-induced oxidative stress. We considered dividing N2a cells into neuronal cells since some cytotoxic properties are not revealed in non-excitable cells, such as undifferentiated neuroblastoma cells [42].

#### 2.3.1. Evaluation of the Potential Neurotoxicity on N2a Cells from Increasing Concentrations of Fer-Ger

**Figure 6:**
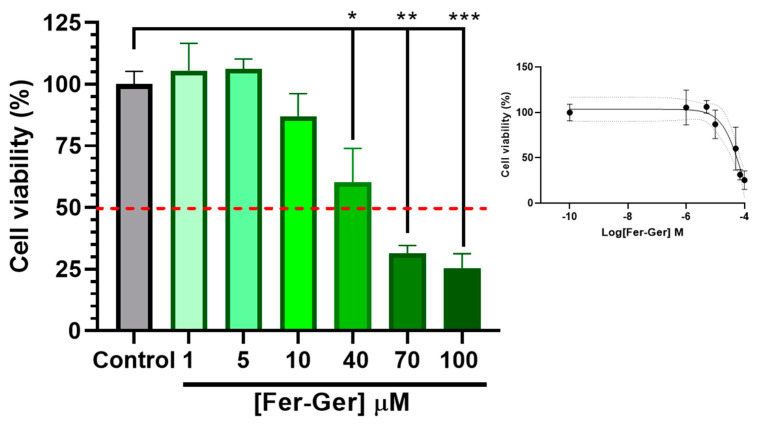
Effect of different concentrations of Fer-Ger on the viability of neuronally differentiated N2a cells. Red line highlights 50% of cell viability (IC50; see the insert). The insertion of the figure reports the sigmoidal plot for IC50 calculations. * *p* < 0.05, ** *p* < 0.001 and *** *p* < 0.0001, mean significant differences compared with the control cells using one-way ANOVA, followed by Dunnett’s multiple comparisons test.

To detect cell viability and cytotoxicity, the MTT (1-(4,5-dimethylthiazol-2-yl)-3,5-diphenylformazan) assay [42] is commonly used. Therefore, to investigate the potential neurotoxic effects of Fer-Ger, increasing concentrations of the compound on neuronal differentiated N2a cell viability were first evaluated by a standard MTT assay; this investigation allowed us to choose the appropriate Fer-Ger concentrations to be used in the following cell uptake studies. The MTT colorimetric method applied to metabolically active cells is based on the reduction in a yellow tetrazolium salt (MTT) to a purple formazan product, whose amounts are proportional to the number of viable cells. As shown in Figure 6, the viability of N2a cells exposed for 1 h to increasing Fer-Ger concentrations (1–100 μM) decreased significantly.

In particular, the comparison with untreated cells revealed a neurotoxic effect of Fer-Ger, starting at 40 μM concentration (*p* < 0.05); following the incubation with the higher 70 μM and 100 μM Fer-Ger concentrations, a trend to further increase in neurotoxicity was observed (*p* < 0.001 vs. the control group), although the Fer-Ger-induced reduction in cell viability was not significantly different from that observed at 40 μM. The IC50 value of cell viability, calculated by the semi-logarithmic plot reported in the inset of Figure 6, was 84.09 ± 0.26 μM. Taking into account the results, Fer-Ger was subsequently applied in N2a cell uptake assays at the low non-toxic concentrations of 5 and 10 μM.

#### 2.3.2. Intracellular Uptake of Fer-Ger

**Figure 7:**
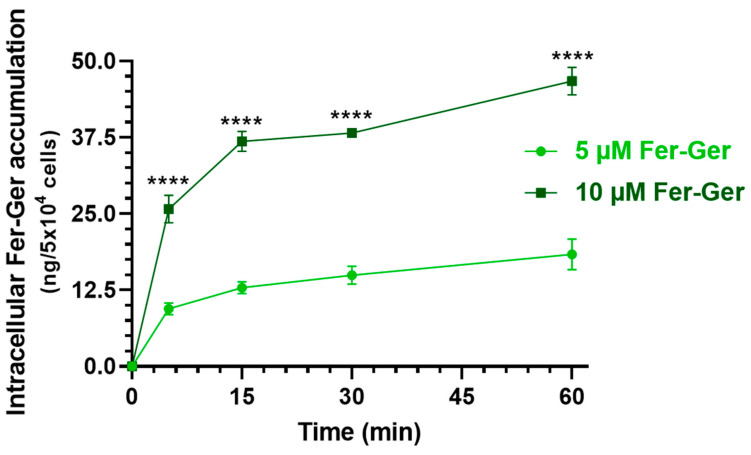
Time-dependent cellular uptake of Fer-Ger by neuronally differentiated N2a cells. N2a cells were incubated with 1 or 5 μM Fer-Ger in DPBS for 1 h at 37 °C. **** *p* < 0.01 comparison of 5 µM Fer-Ger to 10 µM Fer-Ger at each time, using multiple *t*-tests per row by means of the two-stage linear step-up procedure of Benjamini, Krieger, and Yekutieli statistical analysis. Data are reported as mean ± S.D. (*n* = 4) for different experiments.

Considering that Fer-Ger was not hydrolyzed in rat brain homogenate (Section 2.2), we verified whether this conjugate is suitable to be internalized in brain neurons, thus possibly inducing neuroprotective effects by itself. In particular, we considered crucial the finding of internalization and accumulation of Fer-Ger into neuronal differentiated N2a cells to support its potential therapeutic effect in the CNS. It is indeed recognized that intracellular drug concentration can directly reflect drug efficacy and toxicity since the correlation between the drug concentration in the cell and the therapeutic or toxic reaction is stronger than in the plasma [28], where drug concentrations cannot fully elucidate the pharmacological effects of drugs in some tissues, such as tumors or brain tissues [28]. Therefore, the evaluation of the in vitro cell uptake of a compound appears useful to predict drug efficacy in vivo truly and effectively [28]. Neuronal differentiated N2a cells were therefore incubated with Fer-Ger at both the final concentrations of 5 μM and 10 μM in DPBS, for an incubation time course of 5, 15, 30, and 60 min at 37 °C. The relationship between the incubation time and the cellular uptake of Fer-Ger was quantified by measuring, via HPLC, the intracellular concentrations at various time points after cell lysis. The amount of conjugate accumulated in the cells at each time point was normalized to the number of cells in each well. As shown in Figure 7, the uptake profile of 10 μM Fer-Ger in the N2a cells was significantly different and higher than that obtained at 5 μM concentration; in particular, 10 μM Fer-Ger sharply increased within the first 5–15 min of incubation, reaching the intracellular concentration of 36.83 ± 1.65 ng/5 × 10^4^ cells; then its uptake rate into N2a cells decreased, leading to reaching the steady state (46.73 ± 2.25 ng/5 × 10^4^ cells) within 60 min.

On the other hand, 5 μM Fer-Ger increased within the first 5 min of incubation, reaching the intracellular concentration of 9.46 ± 0.98 ng/5 × 10^4^ cells; then its uptake rate into N2a cells decreased, leading to saturation (18.36 ± 2.52 ng/5 × 10^4^ cells) within 60 min. Therefore, these data indicate a concentration- and incubation time-dependent cellular uptake of Fer-Ger by N2a cells, indicating the aptitude of this compound to target brain neuronal cells.

It is noteworthy that no Ger amounts were detected by HPLC analysis, neither in the intracellular nor in the extracellular milieu. This result indicates that the Fer-Ger conjugate is not hydrolyzed by neuronal cells, according to the experimental data obtained in rat brain homogenate.

#### 2.3.3. Fer-Ger Counteracts H_2_O_2_-induced Cell Viability Impairment in N2a Cells

**Figure 8:**
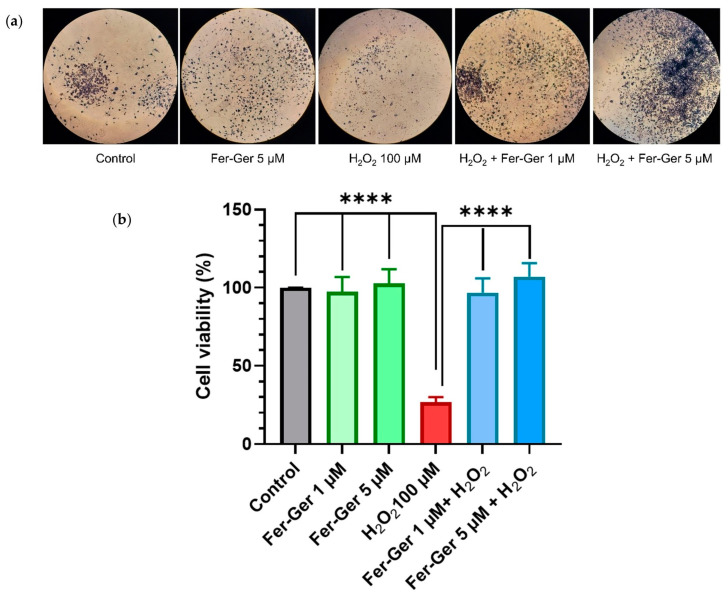
(**a**) N2a cells containing formazan crystals following staining with MTT in different experimental conditions: untreated (Control); treated with 5 µM Fer-Ger (Fer-Ger 5 µM); treated with 100 µM H_2_O_2_ (H_2_O_2_ 100 µM); treated with 100 µM H_2_O_2_ in the presence of 1 µM Fer-Ger (H_2_O_2_ + Fer-Ger 1 µM); treated with 100 µM H_2_O_2_ in the presence of 5 µM Fer-Ger (H_2_O_2_ + Fer-Ger 5 µM). (**b**) Effect of Fer-Ger on H_2_O_2_ induced oxidative stress in N2a cell viability. N2a cells were exposed to 1 and 5 µM Fer-Ger for 1 h followed by a treatment with 100 μM H_2_O_2_ for 24 h. Cell viability was measured by the MTT assay. Data are expressed as mean ± S.E.M. (*n* = 4). **** *p* < 0.0001 H_2_O_2_-treated cells compared to control (untreated cells) and to 1 and 5 µM Fer-Ger-exposed cells; statistical analyses were performed by one-way ANOVA, followed by Dunnett’s multiple comparisons test.

As the beneficial biological effects of Fer and Ger are attributable, at least in part, to their antioxidant properties, it became relevant to verify if their antioxidant behavior is maintained in the Fer-Ger conjugate. To this end, we have verified the potential effect of Fer-Ger in preventing oxidative stress-induced neuronal cell death. Neuron-differentiated N2a cells were therefore preincubated for 1 h with 1 µM and 5 µM Fer-Ger and challenged with the key oxidant H_2_O_2_ (100 µM for 24 h; Figure 8). Cell viability was then monitored using MTT. A highly significant impairment of cell viability, corresponding to a 74% reduction compared to the untreated cells (*p* < 0.0001), was observed in 100 µM H_2_O_2_-treated cells, in agreement with the study of Ghaffari et al. [43], performed on the same cell model. Otherwise, Fer-Ger fully prevented N2a cells from the damage induced by 100 µM H_2_O_2_ at both the concentrations of 1 µM and 5 µM (*p* < 0.0001), demonstrating the neuro-protective effect of this conjugate against H_2_O_2_-induced cytotoxicity in N2a cells.

H_2_O_2_-induced oxidative stress-mediated neuronal cell death is a well-known significant trigger of several neurological disorders, so our results allow us to propose Fer-Ger as a useful agent to prevent neurodegenerative diseases, being the prodrug able to induce neuroprotective effects. To provide a more comprehensive understanding of the neuroprotective profile of Fer-Ger, its potential modulating effect on intracellular ROS production was investigated.

#### 2.3.4. H_2_O_2_ and Fer-Ger Induced ROS Production in Differentiated N2a Cells

**Figure 9:**
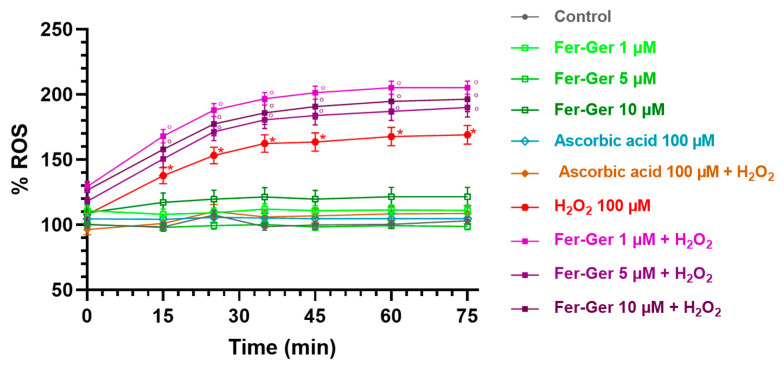
ROS production in differentiated N2a cells exposed to oxidative stress by H_2_O_2_ (100 µM) and treatments with Fer-Ger (1, 5, and 10 µM) or ascorbic acid (100 µM). Data are reported as percentages over control cells, and the assay was carried out for 75 min in order to measure the time-course of intracellular ROS production. Data are expressed as mean ± S.E.M. (*n* = 6). * *p* < 0.05: H_2_O_2_-treated cells compared to the control (untreated cells); ° *p* < 0.05: 1, 5, or 10 µM Fer-Ger-exposed cells in the presence of H_2_O_2_ compared to the control (untreated cells).

Intracellular ROS production was detected using the DCFH-DA (10 µM) assay. As shown in Figure 9, the key oxidant H_2_O_2_ (100 µM) significantly and time-dependently increased DCF fluorescence in the N2a cells, thus suggesting that H_2_O_2_ enhanced ROS production. The maximum increase was reached 40 min after H_2_O_2_ application and was still present at the end of the observation period (75 min).

On the contrary, the application of Fer-Ger (1, 5, and 10 µM) did not affect the ROS production in N2a cells. Interestingly, Fer-Ger, at all the concentrations and times tested, increased the H_2_O_2_-induced ROS production (*p* < 0.05). Finally, these results and assay were validated by applying the reference antioxidant ascorbic acid (vitamin C; 100 µM), which completely prevented the H_2_O_2_-induced ROS production without by itself modifying basal ROS production in N2a cells (Figure 9). These results suggest that Fer-Ger acts, at least under the present experimental conditions, as a pro-oxidant rather than an antioxidant compound. We could therefore hypothesize that the protective effect of Fer-Ger against the H_2_O_2_-induced reduction in N2a cell viability (i.e., MTT results) could be due to the ability of Fer-Ger to induce a hyperactivation of mitochondria, leading to an increased dehydrogenase activity and resulting in an enhanced conversion of tetrazolium salt to MTT formazan also in H_2_O_2_ stressed cells. Similar observations were previously reported by Rai et al. [44] while studying the cell growth inhibitory effects of radiation, like those observed in the literature for polyphenols when using the MTT assay [45]. In addition, mitochondria are known to be responsible for maintaining redox balance and generating energy through oxidative phosphorylation, which in turn is a constant source of ROS production as a side product of the operation of the mitochondrial electron transport chain [46]. Overall, these findings can open future perspectives for investigating Fer-Ger as a new potential mitochondria-targeted anticancer compound against brain tumors such as neuroblastomas or glioblastomas, as extensively reviewed [47,48].

### 2.4. Preparation and Characterization of Tristearin-Based SLMs

The data described in the above sections evidence that Fer-Ger acts as a prodrug of Ger and Fer at the peripheral level, whereas at the central level it can induce neuroprotective effects. Fer-Ger appears, therefore, to be a good candidate to be encapsulated in microcarrier systems to make the most of its beneficial properties. In particular, lipid carriers properly formulated may allow to control its release (potentially prolonging its permanence itself and the permanence of its hydrolysis products at the peripheral level) or to support its targeting toward the CNS after nasal administration, as we have previously demonstrated for several drugs or their prodrugs [25,26,27,49,50].

Fer-Ger-loaded SLMs were obtained by a hot emulsion technique [51,52] using tristearin as lipid material and Tween 60 as emulsifier, in the absence or presence of glucose. The SLMs are considered very simple carriers, and precisely for this reason, their formulation and purification are easy to perform according to procedures that do not require the use of organic solvents. The lipid material used to produce SLMs is biocompatible and biodegradable, so this type of carrier evidences high tolerability in the body, also due to the absence of organic solvents [51]. Moreover, the procedures of SLM formulation can be easily reproducible in the pharmaceutical industry and sustainable for the environment, in line with the enzymatic processes here described for the synthesis of Fer-Ger.

Glucose was proposed as a cryoprotectant for the freeze-drying processes related to the SLMs formulation [53]. This sugar was preferred as a cryoprotectant with respect to mannitol, whose presence in the formulation induced a strong aggregation of the microparticles. We have therefore evaluated the impact of glucose on the prodrug loading on the SLMs and its related release profile.

**Figure 10:**
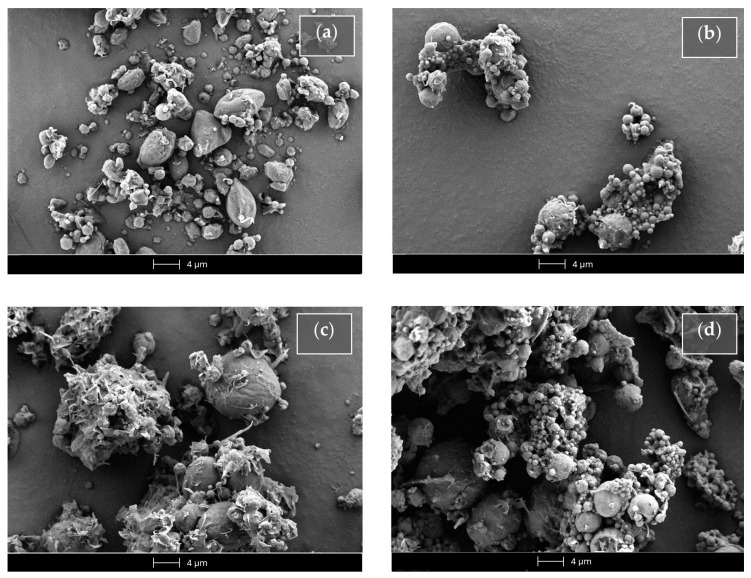
Scanning electron microscopy (SEM) micrographs of the SLMs based on tristearin in the absence (Ts SLMs) or in the presence (Ts-glu SLMs) of glucose. (**a**) Unloaded tristearin-based SLMs. (**b**) Tristearin-based SLMs loaded with Fer-Ger. (**c**) Unloaded tristearin-based SLMs in the presence of glucose. (**d**) Tristearin-based SLMs in the presence of glucose loaded with Fer-Ger.

Figure 10 reports the SEM micrograph of the unloaded or Fer-Ger-loaded tristearin SLMs obtained in the absence or presence of glucose.

The unloaded SLMs obtained in the absence of glucose (Ts SLMs, Figure 10a) evidenced an irregular shape, close to the spherical one, with sizes ranging approximatively between 0.5 and 5 μm. On the other hand, the Fer-Ger loading in the absence of glucose allowed to obtain microparticles (FG Ts SLMs) with a regular spherical shape (Figure 10b) showing similar sizes to those unloaded, even if with some aptitude towards aggregation. The unloaded microparticles formulated in the presence of glucose (Ts-glu SLMs, Figure 10c) evidenced a more regular shape and a slight increased size in comparison to the unloaded SLMs obtained in the absence of the sugar. In Figure 10c, the glucose evidenced a totally irregular shape between the microparticles; its presence induced a weak aptitude for aggregation of the microparticles in the solid sample. The Fer-Ger loading of the SLMs in the presence of glucose (FG Ts-glu SLMs) allowed them to obtain their spherical shape, maintaining the aggregation aptitude in the solid sample (Figure 10d).

The loading values of the SLMs were obtained by HPLC analysis. The amounts of Fer-Ger encapsulated in tristearin-based microparticles obtained in the absence (FG Ts SLMs) or in the presence of glucose (FG Ts-glu SLMs) were 1.461 ± 0.124% *w*/*w* and 1.445 ± 0.058% *w*/*w*, corresponding to encapsulation efficiencies (EE) of 56.19 ± 4.77% and 55.58 ± 2.23%, respectively. These encapsulation values appear significantly higher than those previously obtained for Fer (EE about 14% corresponding to 0.38% *w*/*w*), Fer-Me (EE about 28% corresponding to 0.72% *w*/*w*) [25], Fer-Fer-Me (EE about 19% corresponding to 0.49% *w*/*w*) [26], or the prodrug UDCA-AZT (EE about 26% corresponding to 0.57% *w*/*w*) [50] loaded in the same carriers, suggesting that the adopted formulation conditions optimize the encapsulation of Fer-Ger in the tristearin microparticles, with respect to the other compounds. This Fer-Ger behavior may be attributed to its higher lipophilicity than that of the other compounds. Moreover, our results indicate that the presence of glucose in the SLM formulation procedures did not significantly influence the prodrug loading. On the other hand, the sugar appeared to be able to strongly influence the Fer-Ger release from the SLMs, as described in the following section.

### 2.5. In Vitro Fer-Ger Dissolution and Release from Tristearin-Based SLMs

The dissolution studies of Fer-Ger and its release from SLMs were performed at 37 °C in a mixture of DPBS and MeOH (70:30 *v*/*v*). The MeOH presence was required to ensure sink conditions for Fer-Ger, characterized by very poor water solubility (0.26 ± 0.02 μg/mL in pure water and 0.24 ± 0.01 μg/mL in DPBS). Indeed, the solubility of this prodrug in the mixture of DPBS and MeOH was 8.1 ± 0.3 μg/mL. The addition of MeOH in the release medium constitutes a strategy previously adopted for the release studies from SLMs of compounds characterized by very poor water solubility [26,50].

**Figure 11:**
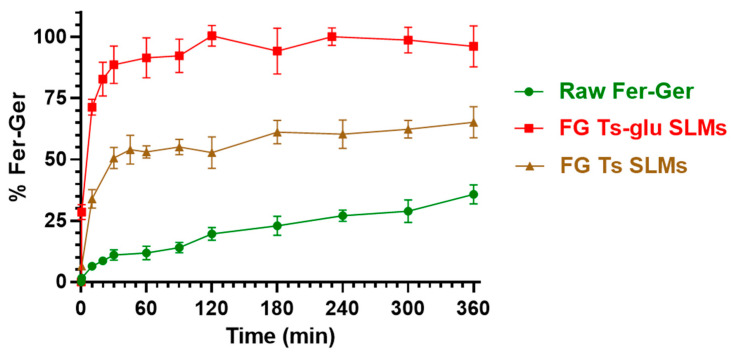
In vitro release of Fer-Ger from tristearin-based SLMs in the absence (FG Ts SLMs) or in the presence (FG Ts-glu SLMs) of glucose in the formulation. The release profiles are compared with the raw Fer-Ger dissolution profile over time. The data are reported as the mean ± S.E.M. of three independent experiments.

Figure 11 reports a comparison of the dissolution profile of the raw compound with its release profile from the loaded tristearin SLMs formulated in the absence of glucose (FG Ts SLMs) or in its presence (FG Ts-glu SLMs).

Fer-Ger was characterized by a weak dissolution rate; in particular, about 30% of the total raw powder was solubilized within 6 h of incubation. Under the same conditions, the release rate of Fer-Ger from the SLMs was significantly higher than the prodrug dissolution rate. Moreover, specific differences in Fer-Ger release rates were observed between the microparticles formulated in the absence or in the presence of glucose. In particular, the samples obtained in the absence of the sugar (FG Ts SLMs) evidenced a release pattern of Fer-Ger characterized by a burst effect of about 30% of the loaded Fer-Ger within 10 min; then, about 50% of the encapsulated prodrug appeared released within 1 h, reaching a maximum of about 60% within 6 h. These data indicate that FG Ts SLMs induce an increase in the dissolution rate of Fer-Ger, allowing at the same time to control its release over time. The ability of these SLMs to increase the dissolution rate of the prodrug may be attributed to the relatively high specific surface area of the particles. This phenomenon appears to be amplified by the SLMs obtained in the presence of glucose. Indeed, the FG Ts-glu SLMs induced a burst effect of about 70% of the loaded Fer-Ger within 10 min, then the complete release of the loaded prodrug was obtained within 2 h. This effect appears to be attributable to the ability of the sugar to enhance the dispersion of particulate systems in aqueous media, thereby increasing their wettability [54,55].

### 2.6. Hydrolysis in Rat Liver Homogenates of Fer-Ger Encapsulated in SLMs

**Figure 12:**
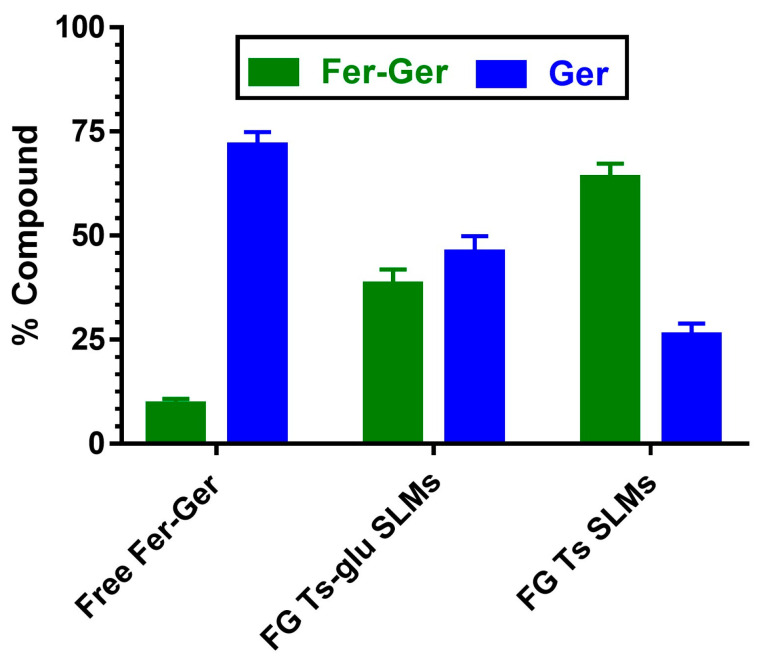
Hydrolysis in rat liver homogenates of free or encapsulated Fer-Ger in SLMs obtained in the absence (FG Ts SLMs) or presence of glucose (FG Ts-glu SLMs). All values are reported as the percentage of the overall amount of incubated prodrug. Data are reported as the mean ± S.E.M. of four independent experiments.

The ability of SLMs to protect and stabilize the prodrug in physiologic environments was analyzed by comparing the hydrolysis rates of free and encapsulated Fer-Ger in rat liver homogenates. This physiologic medium was chosen because it induces a hydrolysis rate of the prodrug higher than that obtained in rat whole blood. As reported in Figure 12, after 10 min of incubation, about 90% of the solubilized free prodrug was hydrolyzed, resulting in the appearance of Ger. The amounts of the prodrug and its hydrolysis product were equivalent to those quantified during the pharmacokinetic study described in Figure 5. The Fer-Ger hydrolysis rate was substantially unaffected when the prodrug was incubated in rat liver homogenates in the presence of the unloaded SLMs. On the other hand, following a 10 min incubation of FG Ts SLMs in rat liver homogenates, about 65% of the prodrug was still present, with correspondingly lower amounts of Ger produced by hydrolysis (about 25%; Figure 12) than those obtained by the incubation of the free prodrug (about 75%; Figure 12). Alternatively, 10 min of incubation of FG Ts-Glu SLMs allowed to quantify about 35% of Fer-Ger still present and about 65% of Ger released by hydrolysis. These results indicate that the ability of the tristearin-based SLMs to stabilize the prodrug in rat liver homogenates is related to their capacity to control the release of the encapsulated Fer-Ger, described in Figure 11.

### 2.7. Limitations and Perspectives

The overall results reported in this work confirm the prodrug approach as a promising strategy to optimize the drug loading in lipid microcarriers of compounds that normally are poorly or not at all encapsulated, such as Fer or Ger, respectively. The synthesis of this prodrug is proposed in an enzymatic way that is able to increase the yield in comparison to previously published methods, even if further optimization of this aspect can be achieved. The ester conjugate Fer-Ger evidences an encapsulation efficiency in tristearin microparticles up to four times higher than that of Fer; moreover, the encapsulation difficulties of Ger, caused by its high volatility [27], appear efficaciously overcome by the prodrug synthesis. Again, the Fer-Ger loading in SLMs offers good versatility in terms of its release patterns and hydrolysis modulation. This study is limited to tristearin microparticles; new encapsulation studies in other carriers could allow further optimization of these aspects. The conjugate Fer-Ger evidences prodrug features at the peripheral level of the body but not at the central level, where, however, it appears potentially able to induce neuroprotection by the retained antioxidant properties of Fer and Ger. This property is, however, limited by a narrow range of Fer-Ger concentrations beyond which neurotoxicity is induced. Another drawback of this study is the discrepancy between the neuroprotective effect expressed by Fer-Ger as a potential radical scavenger on H_2_O_2_-stressed N2a cells in the MTT assay and the pro-oxidant behavior shown by Fer-Ger in the DCFH-DA assay for ROS production in the same cells. However, these findings not only broaden the scope of the study but also provide insights for future investigations into the potential effects of the conjugate against different types of brain cancer. According to our in vitro results, the ability of FG Ts SLMs to control the prodrug release suggests their use for intramuscular or subcutaneous administration to prolong the effects of Fer-Ger and its hydrolysis products both at local and systemic levels. The solid lipid particles are indeed known as drug delivery platforms to optimize the therapeutic effects of drugs [56,57,58]. On the other hand, the neuroprotective effects of Fer-Ger may be potentiated by the nasal administration of the FG Ts-glu SLMs. In particular, their ability to strongly promote the dissolution rate of Fer-Ger may induce its permeation in cerebrospinal fluid across the olfactory mucosa, a phenomenon requiring high concentrations of drugs in the nasal cavity [59]. It is indeed known that appropriate nasal formulations allow for therapeutic amounts of neuroactive drugs in the CNS [26,27,50,60,61]. The loaded Fer-Ger SLMs can therefore be proposed as new “green” formulations potentially able to prolong at the peripheral level of the body the beneficial effects of Fer and Ger or induce neuroprotection in the brain. Finally, this study lacks an in vivo evaluation of Fer-Ger behavior, in particular as far as its pharmacokinetics and potential ability to target the central nervous system are concerned. Further studies of these aspects are therefore necessary to identify the best administration routes and Fer-Ger doses to maximize the potential therapeutic properties of this type of formulation.

## 3. Materials and Methods

### 3.1. Materials

The reagents/substrates for the reaction were *trans*-ferulic acid (99%, Fer) and geraniol (97%, Ger), purchased from Sigma-Aldrich (Milan, Italy). The lipases used were the lipase B from *Candida antarctica* immobilized on a hydrophobic carrier, Lipozyme^®^ 435 (Lipo-435) and the lipase from *Rhizmucor miehei* immobilized on a macroporous anion exchange resin, Lipozyme^®^ RM IM (Lipo-RM). Novozymes SA (Bagsværd, Denmark) has kindly provided the enzymes that showed esterification activity of 56 and 50 U∙g^−1^ for Lipo-435 and Lipo-RM, respectively, determined according to the methodology described by de Oliveira et al. [62]. The *N*-methyl-*N*-(trimethylsilyl) trifluoroacetamide (MSTFA) was purchased from Fluorochem (Hadfield, UK). The solvents dimethyl sulfoxide (DMSO), acetonitrile (CH_3_CN), chloroform, ethyl acetate (Carlo Erba Reagents S.A.S., Milan, Italy), *tert*-amyl alcohol (TAA—Supelco, Milan, Italy), diphenyl ether (DPE—Acros Organics, Geel, Belgium), and deuterated DMSO (Sigma-Aldrich, Milan, Italy) were of analytical grade and used without any additional treatment.

Trizma Base was obtained from Merck Life Sciences Srl (Milan, Italy). Acetonitrile (CH_3_CN), methanol (MeOH), dichlorometane (CHCl_3_), and ethyl acetate were of high-performance liquid chromatography (HPLC) grade from Carlo Erba Reagents (Milan, Italy). The water used for the HPLC analysis was deionized and further purified using Sartorius Arium^®^ Advance EDI (Sartorius Lab Instruments GmbH & Co., KG, Göttingen, Germany). Tristearin, glucose, and Tween 60 were supplied by Merck Life Sciences Srl (Milan, Italy). Dulbecco’s modified Eagle’s medium (DMEM) + Glutamax, phenol-red free DMEM medium, fetal bovine serum (FBS), penicillin, streptomycin, trypsin-EDTA, and Dulbecco’s phosphate buffered saline (DPBS) were furnished by Thermo-Fisher Scientific (Milan, Italy) and Microtech (Naples, Italy). Ethanol, all-trans retinoic acid (atRA), bovine serum albumin (BSA), 2′,7′-dichlorofluorescin diacetate (DCFH-DA), ascorbic acid, and hydrogen peroxide (H_2_O_2_) were obtained from Merck Life Sciences Srl (Milan, Italy). The cell culture vessels were obtained from Thermo-Fisher Scientific (Milan, Italy) and Biosigma (Venice, Italy). The mouse Neuro2a (N2a) neuroblastoma cell line (RRID:CVCL_0470) was purchased from Tebu-bio Srl (Milan, Italy). Male Wistar rats were purchased from Charles River laboratories (Calco, Italy).

### 3.2. Fer-Ger Quantification

The reaction conversion was quantified by taking aliquots of 60 μL for reactions with solvent and 30 μL for solvent-free reactions and removing particulate material by centrifugation (14,000 rpm for 2 min). Afterward, the samples were derivatized by adding 100 μL of MSTFA and warmed for 1.5 h at 60 °C under magnetic stirring (200 rpm). After this time, the samples were diluted with 500 μL of ethyl acetate and injected into a Thermo Focus gas chromatograph (ThermoFisher Scientific, Milan, Italy) equipped with a flame ionization detector (FID) and a MEGA-SE-52 column (30 m length × 0.32 mm internal diameter × 0.1–0.15 µm film thickness, MEGA S.r.l., Legnano, Italy). The heating oven program was 100 °C for 2 min and then 8 °C∙min^−1^ up to 300 °C, remaining at this last temperature for 2 min. The injector and detector temperatures were 175 and 250 °C, respectively. The split injection mode (1:50) was used with an injection volume of 1 μL and H_2_ as the carrier gas. A typical gas-chromatogram of the reaction mixture is reported in the Figure S1 of the Supplementary Material. The conversion of Fer to Fer-Ger was calculated from the ratio between the peak area of Fer-Ger and the sum of the areas of Fer-Ger and Fer (the limiting reagent) [63,64].

### 3.3. Fer-Ger Enzymatic Synthesis

**Figure 13:**
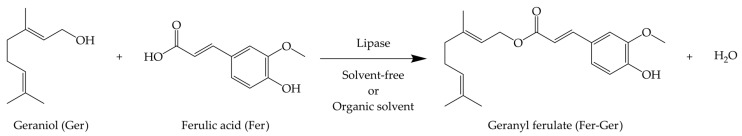
Scheme of the Fer-Ger synthesis through the esterification of Fer with Ger catalyzed by lipase.

The Fer-Ger synthesis through the esterification of Fer with Ger catalyzed by lipases (Figure 13) was carried out in a glass reactor with a concave bottom with a total reaction volume of 13 mL closed hermetically by a screw lid with a Teflon seal under magnetic stirring. The reactor temperature was controlled by immersing the reactor in a thermostatic oil bath with automatic temperature control.

The reactions were performed in a solvent-free system (SF) or in the presence of an organic solvent (DMSO, DPE, TAA, and CH_3_CN) using the commercial lipases Lipo-435 and Lipo-MR as the biocatalysts. The reaction time was 120 h, and the reaction conditions for both systems are presented in Table 1. Blanc experiments without lipases were conducted under the same conditions described in Table 1, and under all the conditions tested, no ester formation was observed in the absence of the biocatalyst.

Subsequently, a kinetic study was conducted to evaluate the reaction progress with the two lipases in a solvent-free system and the presence of DPE using the experimental conditions described in Table 1. After the proper reaction time, aliquots were withdrawn, treated, and analyzed as described in the Fer-Ger quantification section.

### 3.4. Fer-Ger Purification and Characterization

After the removal of the catalyst by filtration, the reaction medium was lyophilized to remove the excess of Ger and the solvent (if present). The residue was chromatographed on silica gel (60 Å, 70–230 mesh, particle size 63–200 μm; Sigma-Aldrich, Milan, Italy) using chloroform/diethyl ether (4:1, *v*/*v*) as the eluent. The fractions corresponding to Fer-Ger were identified by TLC analysis performed on silica gel 60 F250, with detection by carbonization with phosphomolybdic acid solution. The purified product’s ^1^H and ^13^C NMR spectra were acquired at room temperature with a spectrometer operating at 400 MHz (Varian Mercury Plus400, Palo Alto, CA, USA) for the atom numbering, see the Supplementary Material Figures S2 and S3. ^1^H NMR (400 MHz, DMSO) δ 7.51 (d, *J* = 15.9 Hz, 1H, H-7), 7.29 (d, *J* = 2.0 Hz, 1H, H-3), 7.08 (dd, *J* = 8.3, 2.0 Hz, 1H, H-6), 6.75 (d, *J* = 8.2 Hz, 1H, H-5), 6.45 (d, *J* = 15.9 Hz, 1H, H-8), 5.36–5.30 (m, 1H, H-2a), 5.08–5.02 (m, 1H, H-6a), 4.62 (d, *J* = 7.1 Hz, 2H, H-1a), 3.78 (s, 3H, OCH_3_), 2.08–1.94 (m, 4, H-4a and H-5a), 1.67 (s, 3H, CH_3_), 1.61 (s, 3H, CH_3_), 1.54 (s, 3H, CH_3_). ^13^C NMR (101 MHz, DMSO) δ 167.03, 149.86, 148.37, 145.45, 141.82, 131.53, 125.95, 124.19, 123.62, 119.16, 115.91, 114.88, 111.56, 60.83, 56.09, 39.39, 26.24, 25.95, 18.02, 16.64. The mass of Fer-Ger has been determined as described in the Supplementary Material (Figure S4 and Table S1): HRMS (ESI) *m*/*z* calcd for C_20_H_27_O_4_^+^: 331.1904 [M + H]^+^; found: 331.1903.

### 3.5. Stock Solution of Fer-Ger and the Compounds Derived by Its Hydrolysis

Gerand Fer-Ger were dissolved in DMSO at the final concentration of 5 × 10^−2^ M to obtain stock solutions, which were stored at −20 °C until their experimental use.

### 3.6. Kinetic Analysis in Whole Blood

Kinetic analysis of Fer-Ger was performed by incubating the compound at 37 °C in heparinized whole blood obtained from healthy human volunteers or from male Wistar rats weighing 200–250 g. In particular, appropriate amounts of stock solution in DMSO were added to a volume of 3 mL of whole blood in order to obtain the final concentration of 30 µM (9.92 µg/mL). Samples (100 μL) were withdrawn at defined time intervals and immediately treated. Specifically, they were firstly added to ice-cold water (500 µL); then, sulfosalicylic acid (10%, 50 µL) and the internal standard (100 μM carbazole dissolved in a mixture of MeOH and H_2_O 50:50 *v*/*v*, 50 µL) were added; finally, 1 mL of H_2_O-saturated ethyl acetate was used twice as extraction solvent. The samples were centrifuged (13,500× *g* for 10 min) and the organic phase was evaporated under a nitrogen stream. The residue was re-suspended in a mixture of H_2_O and CH_3_CN (50:50 *v*/*v*, 150 µL) and centrifuged (16,000× *g* for 5 min). The treated samples (15 µL) were injected into the HPLC-DAD apparatus (see below) for the quantification of Fer-Ger and its potential hydrolysis product, Ger. All the values were derived from the mean of three independent incubation experiments.

### 3.7. Preparation of Rat Liver Homogenates

Livers were obtained from male Wistar rats, which were sacrificed by decapitation, after a slight isoflurane anesthesia. The livers were immediately isolated, and, after washing with ice-cold saline solution, they were homogenized in 4 volumes (*w*/*v*) of Tris HCl (50 mM, pH 7.4, 4 °C) by using a Potter–Elvehjem apparatus. The supernatant obtained after centrifugation (2000× *g* for 10 min at 4 °C) was decanted off and stored at −80 °C before its use for kinetic studies. The total protein concentration in the tissue homogenate was determined using the Lowry procedure [65] and resulted in 30.6 ± 1.1 μg protein/μL.

### 3.8. Preparation of Rat Brain Homogenates

Brains were obtained from male Wistar rats, which were sacrificed by decapitation after a slight isoflurane anesthesia. The brains were immediately separated and homogenized in 5 volumes (*w*/*v*) of Tris HCl (50 mM, pH 7.4, 4 °C) using an ultra-Turrax (IKA Werke GmbH & Co. KG, Staufen, Germany) with 3 × 15 s bursts. After centrifugation of the homogenate (3000× *g* for 15 min at 4 °C), the supernatant was isolated and stored at −80 °C until its use for in vitro kinetic studies. The Lowry procedure [65] was adopted to quantify the total protein concentration in the brain homogenate, resulting in 7.4 ± 0.4 μg protein/μL.

### 3.9. Kinetic Analysis in Tris-HCl

Kinetic analysis of Fer-Ger was performed by its incubation at 20 µM (6.61 µg/mL) final concentration and at 37 °C in 30 mL of a mixture of Tris-HCl buffer 50 mM (pH 7.4) and MeOH 70:30 (*v*/*v*). The presence of MeOH assured the complete dissolution of Fer-Ger, which is characterized by very poor water solubility (0.77 ± 0.02 μM, corresponding to 0.26 ± 0.01 μg/mL). At defined time intervals, 150 μL of the solution were withdrawn and filtered (regenerate cellulose, 0.45 μm), then 10 µL were injected into the HPLC-DAD apparatus (see below) for the quantification of Fer-Ger and its potential hydrolysis product Ger. All the values were derived from the mean of three independent incubation experiments.

### 3.10. Kinetic Analysis in Rat Brain and Liver Homogenates

Kinetic analysis of Fer-Ger was performed by its incubation at 37 °C in 3 mL of rat liver or brain homogenates at 30 µM (9.92 µg/mL) final concentration. At defined time intervals, 100 μL of homogenates were withdrawn and immediately treated by quenching them in EtOH (250 µL), then an internal standard (100 μM carbazole dissolved in EtOH, 50 µL) was added. The samples were centrifuged (13,500× *g* for 10 min), and the 300 μL aliquots were reduced to dryness under a nitrogen stream. The residue was re-suspended in a mixture of H_2_O and CH_3_CN (50:50 *v*/*v*, 150 µL) and centrifuged twice (16,000× *g* for 5 min), then injected (15 μL) into the HPLC apparatus (see below) for the quantification of Fer-Ger and its potential hydrolysis product Ger. All the values were derived from the mean of three independent incubation experiments.

### 3.11. In Vitro Kinetic Calculations

The Fer-Ger degradation in whole blood or homogenates was analyzed as exponential decay plots of its concentrations versus incubation time, and the related half-life values were obtained from the linear regression of the corresponding semi-logarithmic plots. The quality of the fits was evaluated by considering the correlation coefficients (*r*) and *p* values. The computer program GraphPad Prism 7 (GraphPad, San Diego, CA, USA) allowed to perform the calculations.

### 3.12. HPLC Analysis

Fer-Ger and its hydrolysis product, Ger, were quantified by HPLC. The chromatographic apparatus consisted of a modular system (model LC-40D) pump and DAD detector (model SPD-M40, Shimadzu, Kyoto, Japan) and an injection valve with a 20 μL sample loop (model 7725; Rheodyne, IDEX, Torrance, CA, USA). The analysis of Ger and Fer-Ger was performed by using a 5 μm Hypersil BDS C-18 column (150 mm × 4.6 mm i.d.; ThermoFisher Scientific SpA Italia Srl, Milan, Italy) equipped with a guard column packed with the same Hypersil material; the mobile phase consisted of an isocratic mixture of water and acetonitrile at a ratio of 40:60 (*v*/*v*); the flow rate was 1 mL/min. The chromatograms were displayed at 320 nm wavelength to evaluate the absorbance of Fer-Ger and at 210 nm wavelength to evaluate the absorbance of both Ger and carbazole, this last used as internal standard for the quantification of Ger and Fer-Ger in the samples of blood, or liver and brain homogenates. The retention times of Ger, carbazole, and Fer-Ger were 3.8 min, 4.4 min, and 13.0 min, respectively.

Data acquisition and processing were performed on a personal computer using LabSolutions Software (version 5.110 in Windows 10, Shimadzu, Kyoto, Japan).

The chromatographic precision was evaluated by repeated analysis (*n* = 6) of the same sample solution (10 µL) containing every single compound at a concentration of 10 μM (1.54 μg/mL for Ger; 3.30 μg/mL for Fer-Ger). The compounds were dissolved in a mixture of H_2_O and CH_3_CN (50:50 *v*/*v*), or in a mixture of Tris-HCl buffer 50 mM (pH 7.4) and MeOH (70:30 *v*/*v*), or in a mixture of DPBS and MeOH (70:30 *v*/*v*), or in MeOH, and the chromatographic precision was represented by relative standard deviation (RSD) values ranging from 0.87 to 0.94. Calibration curves of peak areas versus concentration in the range from 0.1 to 50 μM for each compound (0.015–7.71 μg/mL for Ger; 0.033–16.52 μg/mL for Fer-Ger) dissolved in the same media described above were generated and resulted linear in the considered range (*n* = 8, *r* ≥ 0.993, *p* < 0.0001).

In order to avoid interferences during the analysis of the compounds from biological samples, a preliminary analysis was performed on blank blood and rat brain and liver homogenate samples, evidencing that the components of these biological systems do not interfere with the retention times of Ger, Fer-Ger, and the internal standard (carbazole).

The peak areas derived from the extraction of blood test samples (10 μM) at 4 °C (*n* = 6) were compared to those obtained by the injection of solutions of the analytes dissolved in a mixture of H_2_O and CH_3_CN (50:50 *v*/*v*) at the same concentration, and the average recoveries ± SD of Ger and Fer-Ger ranged about from 38% to 58%. A similar analysis was performed for the extraction of Fer-Ger and Ger from rat liver and brain homogenates; the average recoveries ± SD of these compounds ranged from 35% to 86%. Therefore, the concentrations of these compounds determined in whole blood or homogenates were referred to as peak area ratios with respect to the internal standard (carbazole). Applying this method, eight different concentrations in whole blood or rat brain homogenate at 4 °C, ranging from 0.5 to 50 µM for Ger (0.077 to 7.71 µg/mL) and Fer-Ger (0.167 to 16.52 µg/mL), were used to generate calibration curves in these biological systems, which resulted linear (*n* = 8, *r* ≥ 0.992, *p* < 0.001).

### 3.13. N2a Cell Culture and Neuronal Differentiation

N2a cells were seeded on T75 flasks and grown in Dulbecco’s Modified Eagle Medium (DMEM) containing Glutamax, supplemented with 100 μg/mL streptomycin, 100 IU/mL penicillin, and 10% fetal bovine serum (FBS) in a humidified 5% CO_2_ atmosphere at 37 °C. After two passages by trypsinization, cells were seeded at a density of 5 × 10^3^ cells/well in 12-well plates with 2 mL of DMEM or at a density of 5 × 10^2^ cells/well in 96-well plates with 0.2 mL of DMEM, depending on the study (see below). For neural differentiation, N2a cells were allowed to adhere and proliferate in wells for 4 days; then, after washing once with serum-free DMEM, differentiating medium composed of DMEM containing 1% HS and 20 μM all-trans-retinoic acid (atRA) was added in both the seeding conditions, up to 6 days of differentiation, after which the cells were used for the experiments.

### 3.14. MTT Assay for Evaluation of Fer-Ger Toxicity

The potential neurotoxicity of Fer-Ger in N2a differentiated cells, seeded at a density of 5 × 10^2^ cells/well in a 96-well plate as reported above, was assayed with the 3-(4,5-dimethylthiazol-2-yl)-2,5-diphenyltetrazolium (MTT) test after incubation for 1 h at 37 °C in a humidified 5% of CO_2_ atmosphere at 37 °C with increasing concentrations (1, 5, 10, 40, 70, 100 µM) of Fer-Ger dissolved in 0.2 mL of DPBS, supplemented with 0.9 mM CaCl_2_, 0.5 mM MgCl_2_, 5.3 mM glucose, and 0.1% BSA to ensure the solubilization of the compound in aqueous media. After 1 h, 20 µL of MTT (5 mg/mL) in DPBS solution were added to each well (4 h; 37 °C and 5% CO_2_). After the conversion of the substrate to a chromogenic product by metabolically active cells, the MTT solution was removed, and the purple MTT formazan crystals were solubilized with 0.1 mL/well of DMSO for 1 h at 37 °C in an orbital shaker incubator. Finally, the absorbance of each well was measured at 570 nm using a microtiter plate reader (Sunrise^®^ Microplate Reader, Tecan Trading AG, Männedorf, Switzerland). The values were expressed as cell vitality percentages with respect to control in the absence of compounds. Each reported value represents the mean of four independent incubation experiments.

### 3.15. Time-Course of Fer-Ger Uptake in Neuronal Differentiated N2a Cells

The potential ability of Fer-Ger to enter the neuronal cells of the brain, where it may exert its neuroprotective effects, was evaluated in 9 days-neuronal differentiated N2a cells (5 × 10^3^ cells/well in a 12-well plate) at its best non-toxic concentrations of 5 µM and 10 µM in MTT. For their optimal dissolution, 5 µM and 10 µM Fer-Ger solutions in DPBS containing 0.1% BSA were kept for 30 min at 37 °C before the uptake experiment. Each of the 12-well plates was rinsed once with 1 mL of DPBS, and then 1 mL of 5 µM and 10 µM Fer-Ger was added to the test wells, and the plate was incubated for pre-determined interval times (5, 15, 30, and 60 min) in a humidified 5% CO_2_ atmosphere at 37 °C. The incubation was stopped at each time by taking the incubation DPBS from each well and storing it in Eppendorf tubes. Then, 300 µL of MeOH were added to 700 µL of incubation DPBS to ensure complete dissolution of Fer-Ger, and the resulting solution (made of a mixture of PBS/MeOH 70:30 *v*/*v*) was filtered (regenerate cellulose, 0.45 µm) and injected in the HPLC-DAD system (10 µL) for the quantification of extracellular Fer-Ger and its potential hydrolysis product Ger. The well-adherent cells were then lysed by adding 0.2 mL of chilled bi-distilled water, kept at −80 °C for at least 30 min, finally thawed, transferred in Eppendorf tubes where 0.2 mL of CH_3_CN were added, and the resulting suspension (made of a mixture of water and acetonitrile 50:50 *v*/*v*) was centrifuged (16,000× *g* for 10 min) and immediately injected into the HPLC apparatus (10 µL) to quantify the intracellular Fer-Ger and its potential hydrolysis product Ger. The final number of cells was evaluated on untreated wells following their trypsinization and counted using the Scepter^TM^ 2.0 automated cell counter (Merck Millipore, Milan, Italy). Each reported value represents the mean of four independent incubation experiments.

### 3.16. Effects of Fer-Ger on H_2_O_2_-Altered Viability in Differentiated N2a Cells

The MTT assay was also carried out to determine the neuroprotective effect of Fer-Ger on H_2_O_2_-induced cell oxidative stress in N2a neuronal cells [43]. Briefly, N2a cells (5 × 10^2^) were seeded and differentiated into 96-well plates, as reported above. Then, cells were incubated with 1 µM and 5 µM Fer-Ger dissolved in DMEM containing 0.5% HS for 1 h in a humidified 5% CO_2_ atmosphere at 37 °C. The experimental design therefore included cells incubated with only culture medium as a control and cells incubated for 24 h with 100 µM H_2_O_2_ as a damage-inducing agent after 1 h of preincubation in the absence or presence of 1 µM or 5 µM Fer-Ger. Then, 20 μL of MTT (5 mg/mL) was added into the 200 µL of the incubation medium. Four hours later, the medium containing MTT was aspirated and replaced by 100 µL of dimethyl sulfoxide (DMSO) solubilization agent for 1 h at 37 °C in an orbital shaker incubator. Finally, the absorbance of each well was measured at 570 nm using a microtiter plate reader (Sunrise^®^ Microplate Reader, Tecan Trading AG, Männedorf, Switzerland).

### 3.17. Detection of Reactive Oxygen Species (ROS) in Differentiated N2a Cells Treated with Fer-Ger and Stressed with H_2_O_2_

The potential antioxidant effect of Fer-Ger was evaluated according to the original method of Wang and Joseph [66]. In this method, the nonpolar 2′,7′-dichlorofluorescin diacetate (DCFH-DA) enters the intact cells and is hydrolyzed by intracellular esterases to nonfluorescent DCFH carboxylate anion, which remains entrapped and becomes the high fluorescent dichlorofluorescin (DCF) after oxidation by free radicals generated by a stressor, such as H_2_O_2_. Therefore, H_2_O_2_-induced intracellular ROS production was measured in N2a cells by a florescence intensity assay following exposure to the oxidation-sensitive dye DCFH-DA. N2a cells were seeded at a density of 1 × 10^4^ cells/well in a black-coated and clear bottom 96-well plate and neuron-differentiated as reported above. After 6 days, differentiating medium in N2a cells was replaced with pre-warmed phenol-red-free and serum-free DMEM containing 10 µM DCFH-DA and incubated for 30 min in the dark in a humidified 5% CO_2_ atmosphere at 37 °C. The 10 µM DCFH-DA loading solution was then removed, and the cells were washed twice with fresh phenol-red-free and serum-free DMEM to remove the redundant extracellular DCFH-DA probe. Subsequently, N2a cells were incubated with pre-warmed phenol-red free DMEM containing 1% FBS as an untreated control and with solutions dissolved in phenol-red free DMEM containing 1% FBS of Fer-Ger (1, 5, or 10 µM) or 100 µM ascorbic acid (AA, as an established antioxidant positive control) and incubated for 1 h in a humidified 5% CO_2_ atmosphere at 37 °C. After this time, 100 µM H_2_O_2_ was added in the absence or presence of 1, 5 or 10 µM Fer-Ger or 100 µM AA, and the fluorescence intensity was measured at an excitation wavelength of 485 nm and at an emission wavelength of 530 nm, monitoring the oxidation of DCFH to DCF by the intracellular ROS by running the kinetic over 75 min in a fluorescence microplate reader (VICTOR^3^_TM_ 1420 Multilabel counter, PerkinElmer, Milan, Italy). Results were represented as a percentage of intracellular ROS production (100% of control) resulting from the mean values ± SEM of sextuplicate wells over time.

### 3.18. Preparation of Fer-Ger-Loaded Microparticles

Tristearin-based SLMs loaded with Fer-Ger were prepared using the melt oil/water emulsification technique [51,52], via an inversion phase procedure (aqueous phase poured into the molten lipid) in order to avoid loss of drug and excipient during the formulation of the particles. The hot (75–85 °C) deionized H_2_O (18.75 mL) with the surfactant (0.7% *w*/*w*) Tween 60 was poured into the molten lipid phase (1.125 g of tristearin), in which Fer-Ger (30 mg) had been dispersed. The two phases were high-shear mixed (21,500 rpm for 2 min) using an Ultra-Turrax T25 mixer (IKA-Werk, Staufen, Germany) at 75–85 °C, and the obtained emulsion was rapidly cooled to 10 °C using an ice bath under magnetic stirring to obtain a microparticulate suspension that was centrifuged (10,000× *g* for 15 min) to recover the SLMs, which were lyophilized for 24 h to give water-free microparticles. The SLMs were prepared in the absence (FG Ts SLMs) and in the presence (FG Ts-glu SLMs) of glucose in the formulation, added before the centrifugation step in an amount equivalent to 15% *w*/*w* of the lipid phase (0.169 g). Unloaded SLMs, both in the absence (Ts SLMs) and in the presence (Ts-glu SLMs) of glucose, were also prepared with the same procedure by omitting the drug.

### 3.19. Microparticles Characterization

The morphology of the microspheres was obtained using a scanning electron microscope equipped with a lanthanum hexaboride (LaB_6_) emitter (HV-SEM; Zeiss EVO40XVP, Arese, Milan, Italy). The microparticulate formulations were placed on double-sided tape that had previously been secured to aluminum stubs and then analyzed at 20 kV acceleration voltage after gold sputtering.

### 3.20. Fer-Ger Content in the SLMs

The microparticulate powders were characterized in terms of Fer-Ger amounts entrapped in the lipid matrix based on an established method [50]. Briefly, about 5 mg of microparticles were accurately weighed by means of a high precision analytical balance (d = 0.01 mg; Sartorius, model CP 225D, Goettingen, Germany) and dissolved in 2 mL of MeOH at 80 °C for 15 min. The samples were cooled to room temperature, and fresh MeOH was added to reach a final volume of 2 mL. After the filtration of the samples (regenerate cellulose, 0.45 µm) and their dilution 1:10, a volume of 10 µL of the diluted samples was injected into the HPLC-DAD system to quantify Fer-Ger. The drug loading and entrapment efficiency were calculated according to the following Equations (1) and (2):

(1) $$\mathrm{Drug}\;\mathrm{loading}\;(W/W\;\%)=\frac{\mathrm{amount}\;\mathrm{of}\;\mathrm{drug}\;\mathrm{in}\;\mathrm{microparticles}}{\mathrm{amount}\;\mathrm{of}\;\mathrm{loaded}\;\mathrm{microparticles}}\times 100$$

(2) $$\mathrm{Entrapment}\;\mathrm{efficiency}\;(\%)=\frac{\mathrm{amount}\;\mathrm{of}\;\mathrm{drug}\;\mathrm{in}\;\mathrm{microparticles}}{\mathrm{starting}\;\mathrm{amount}\;\mathrm{of}\;\mathrm{drug}}\times 100$$

The data were determined from the average of three independent experiments.

### 3.21. Fer-Ger Solubility, In Vitro Dissolution Rate and Release from the Microparticles

Solubility measurements of pure Fer-Ger were performed by adding an excess amount of Fer-Ger (about 10 mg) in 5 mL of pure water, DPBS, or a mixture made of DPBS and methanol (70:30 *v*/*v*) at 25 °C. The samples were magnetically stirred for 48 h. The supernatant was filtered through a 0.45 μm regenerate-cellulose membrane filter, and 10 µL of the filtered samples were injected into the HPLC-DAD system for Fer-Ger quantification. The study was performed in triplicate.

Fer-Ger dissolution and release from the SLMs were evaluated by adding raw Fer-Ger (about 0.1 mg) or SLMs, containing an equivalent amount of Fer-Ger, to 60 mL of a mixture of DPBS and methanol (70:30 *v*/*v*). The samples were kept under mechanical stirring at 50 rpm and 37 °C. At defined time intervals, 0.2 mL aliquots of the medium were withdrawn and replaced with an equal volume of fresh fluid. The aliquots were filtered (0.45 μm) and injected (10 μL) into the HPLC apparatus for Fer-Ger quantification. The dissolution or release (%) of Fer-Ger was calculated from its total amount incubated as a free compound or contained in the SLMs, respectively. The data were determined from the average of three independent experiments.

### 3.22. Hydrolysis in Rat Liver Homogenates of Fer-Ger Encapsulated in SLMs

Hydrolysis studies of the microencapsulated Fer-Ger were performed in 3 mL of rat liver homogenates at 37 °C with loaded SLMs, by adding the FG Ts-glu SLMs or FG Ts SLMs in order to obtain a Fer-Ger final concentration of about 30 μM (9.92 µg/mL). Since SLMs were suspended in the medium, it was difficult to withdraw several homogeneous samples, and consequently, stability was assessed by a single sampling after 10 min of incubation. Dichloromethane (1 mL) was added, and 250 μL was withdrawn from the samples and quenched in 500 μL of ice-cold ethanol added with 100 μL of internal standard (100 μM carbazole dissolved in ethanol). After 5 min of centrifugation at 16,000× *g*, 600 μL aliquots were reduced to dryness under a nitrogen stream. The obtained residue was re-suspended in 300 μL of the water−acetonitrile mixture (50:50 *v*/*v*) and, after centrifugation, 15 μL was injected into the HPLC system for Ger and Fer-Ger quantification. The same procedure was adopted for free Fer-Ger by adding appropriate amounts of stock solution in DMSO to a volume of 3 mL of rat liver homogenates in order to obtain the final concentration of 30 µM (9.92 µg/mL). In this case, the Fer-Ger hydrolysis was analyzed both in the absence or in the presence of unloaded Ts SLMs or Ts-glu SLMs, whose amounts were equivalent to those of their parent-loaded FG Ts SLMs or FG Ts-glu SLMs, respectively. All the values obtained are the means of four independent experiments.

### 3.23. Statistical Analysis

Statistical analyses on MTT assay for the evaluation of the cytotoxic effects of Fer-Ger (Section 3.14), its protective role on cell viability in H_2_O_2_-damaged cells (Section 3.16) and the DCFH-DA assay (Section 3.17) were performed by one-way analysis of variance (ANOVA), followed by Dunnett’s test of multiple comparisons. Statistical analyses on intracellular content in uptake studies on N2a cells (Section 3.15) were performed by multiple *t*-tests per row by means of the two-stage linear step-up procedure of Benjamini, Krieger, and Yekutieli statistical analysis. All analyses were performed using the computer program GraphPad Prism version 7.0 (GraphPad Software, San Diego, CA, USA), and the significance was set at *p* < 0.05.

## Figures and Tables

**Table 1:** Experimental conditions used for the enzymatic synthesis of Fer-Ger.

	Lipase	Biocatalyst (mg)	Molar Ratio ^1^ (mg/mg)	Temperature (°C)	Agitation (rpm)
Solvent-free	Lipo-435	38	1:20 (50/794.3)	90	500
	Lipo-RM			80	
Solvent (6 mL)	Lipo-435	38	1:06 (50/238.3)	90	500
	Lipo-RM			80	

^1^ Molar ratio Fer to Ger.

## Data Availability

Data are contained within the article and Supplementary Materials.

## Supplementary Materials

The following supporting information can be downloaded at: https://www.mdpi.com/article/10.3390/ijms25116263/s1.
