# Supplementary material for: Characterization and Hydrolysis Studies of a Prodrug Obtained as Ester Conjugate of Geraniol and Ferulic Acid by Enzymatic Way
Source: Int J Mol Sci. 2024 Jun 6;25(11):6263. doi: 10.3390/ijms25116263 (PMC11172460; doi:10.3390/ijms25116263)
Supplement: Supplementary file 1 [file ijms-25-06263-s001.zip › ijms-3012260-supplementary.pdf]

International Journal of *Molecular Sciences*

MDPI

Article

Characterization and Hydrolysis Studies of a Prodrug Obtained as Ester Conjugate of Geraniol and Ferulic Acid by Enzymatic Way

Lindomar Alberto Lerin [1,†], Giada Botti [1,2,†], Alessandro Dalpiaz [1,*], Anna Bianchi [1], Luca Ferraro [3], Chaimae Chaibi [1], Federico Zappaterra [1], Domenico Meola [1], Pier Paolo Giovannini [1] and Barbara Pavan [2,4]

[1] Department of Chemical, Pharmaceutical and Agricultural Sciences, University of Ferrara—UNIFE, Via Luigi Borsari, 46, I-44121 Ferrara, Italy; lrnldm@unife.it (L.A.L.); bttgdi@unife.it (G.B.); bna@unife.it (A.B.); chaimae.chaibi@edu.unife.it (C.C.); zppfrc@unife.it (F.Z.); domenico.meola@unife.it (D.M.); gvnppl@unife.it (P.P.G.)
[2] Center for Translational Neurophysiology of Speech and Communication (CTNSC@UniFe), Italian Institute of Technology (IIT), Via Fossato di Mortara 19, I-44121 Ferrara, Italy; pvnbbr@unife.it
[3] Department of Life Sciences and Biotechnology, University of Ferrara and LTTA Center, Via Fossato di Mortara 19, I-44121 Ferrara, Italy; frl@unife.it
[4] Department of Neuroscience and Rehabilitation—Section of Physiology, University of Ferrara,Via L. Borsari 46, I-44121 Ferrara, Italy
* Correspondence: dla@unife.it
† These authors contributed equally to this work.

Supplementary material

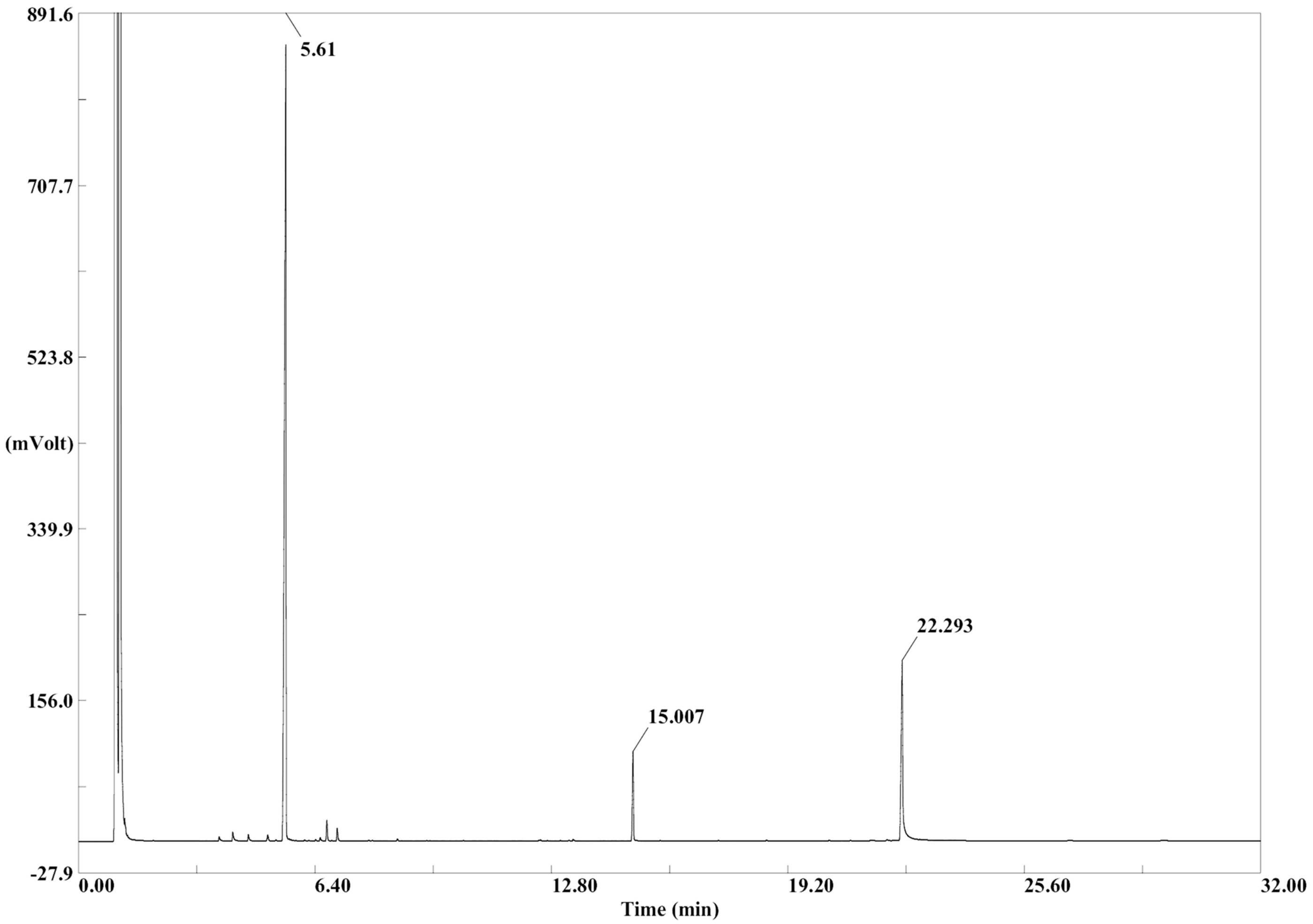

Figure S1. Typical gas-chromatogram of the reaction mixture for the geranyl ferulate synthesis. Retention time: geraniol 5.6 min, ferulic acid 15.0 min and geranyl ferulate 22.2 min.

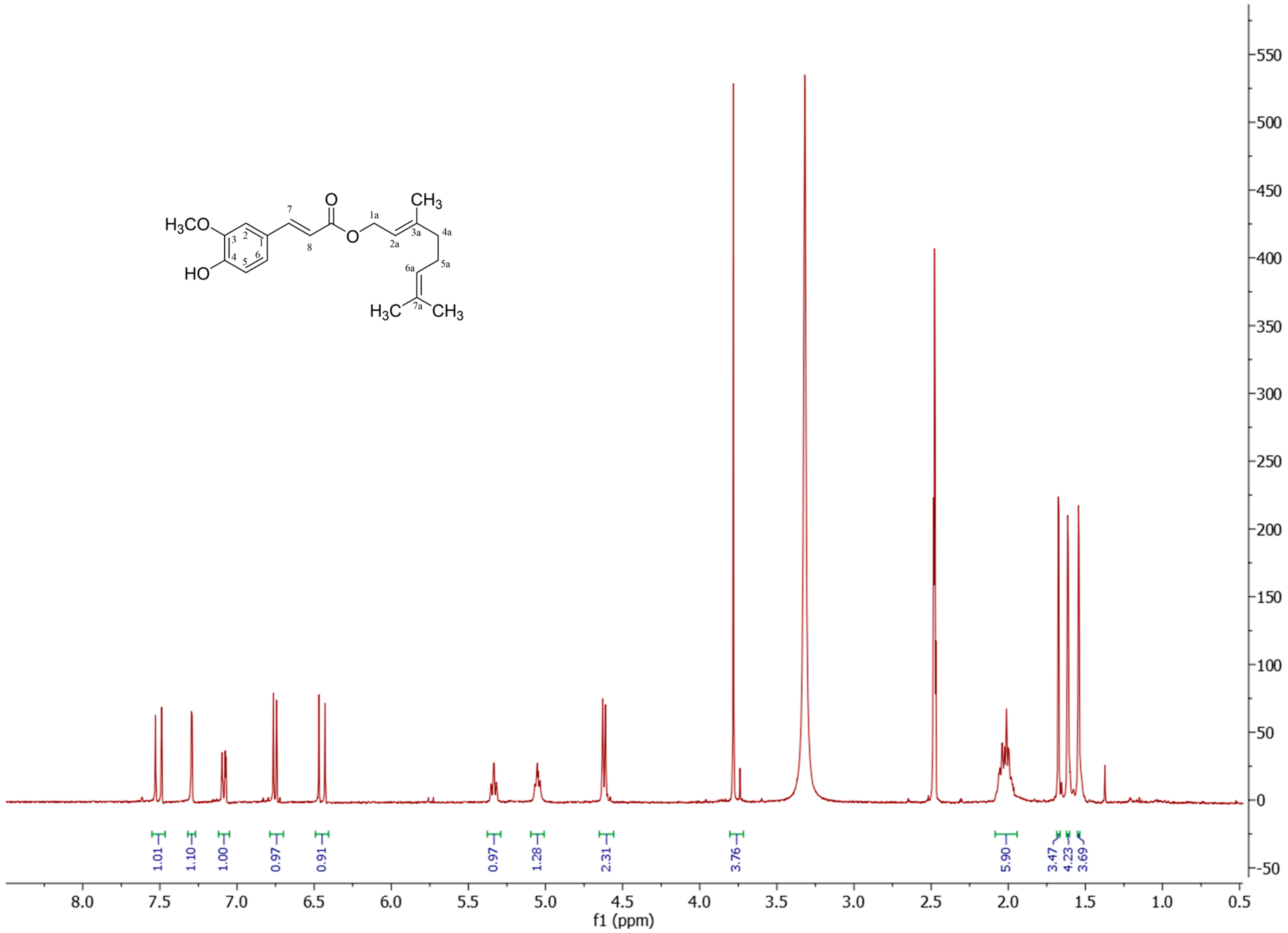

Figure S2. ^{1}H NMR spectrum of geranyl ferulate in DMSO-d_6 (400 MHz).

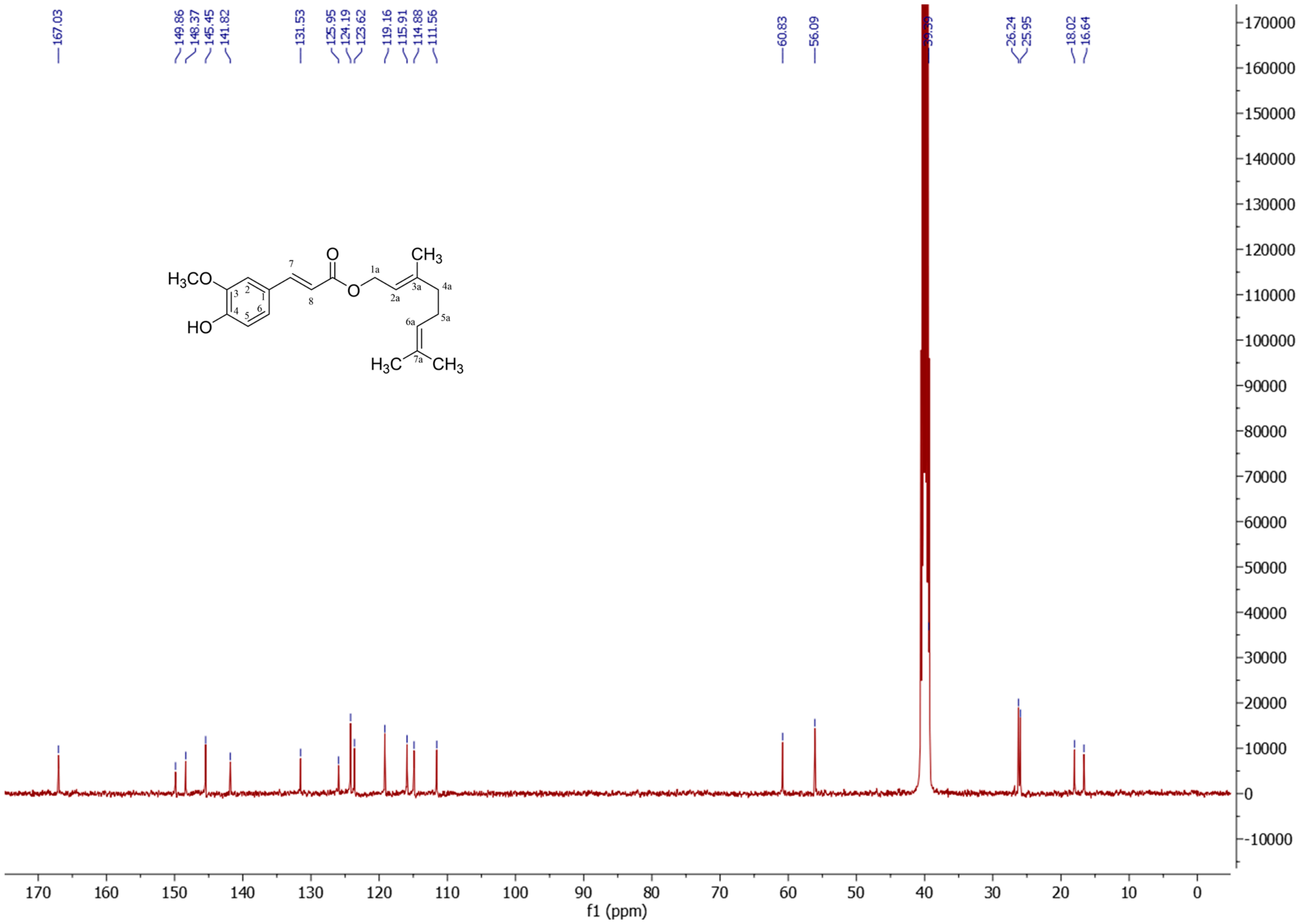

Figure S3. ^{13}C NMR spectrum of geranyl ferulate in DMSO-d_6 (400 MHz).

Mass spectrometry

The mass of the synthesized compound was assessed by injecting 1 µL of sample into a Vanquish Flex Ultra High-Performance Liquid Chromatography (UHPLC) system coupled to a High-Resolution Orbitrap Exploris 240 mass spectrometer (Thermo Fisher Scientific, Milan, Italy). The separation was performed on a Waters BEH C18 column (100×2.1 mm L×I.D., 1.7 µm) operated under reversed phase conditions using water and acetonitrile + 0.1% formic acid as mobile phase. The sample was analyzed in positive mode.

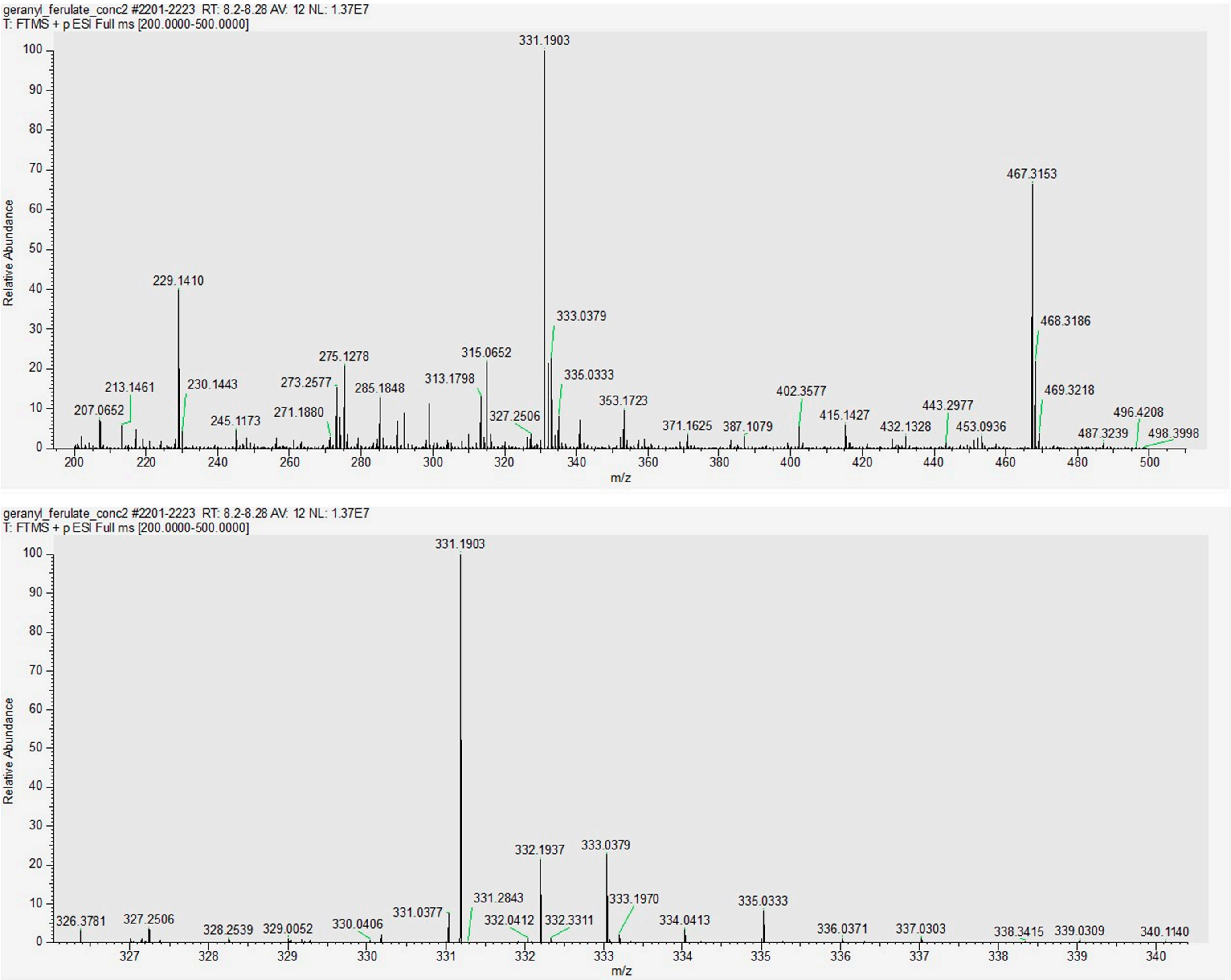

Figure S4. High-Resolution Mass (HRM) spectra of the geranyl ferulate.

Table S1. The calculated and theoretical molecular mass of geranyl ferulate.

Sample	Calculated m/z	Theoretical m/z [M-H]$^+$	Δ (ppm)
**Geranyl Ferulate**	331.1903	331.19039	0.27
